# Supplementary material for: Mechanism of membrane-curvature generation by ER-tubule shaping proteins
Source: Nat Commun. 2021 Jan 25;12:568. doi: 10.1038/s41467-020-20625-y (PMC7835363; doi:10.1038/s41467-020-20625-y)
Supplement: Supplementary file 1 — Supplementary Information [file 41467_2020_20625_MOESM1_ESM.pdf]

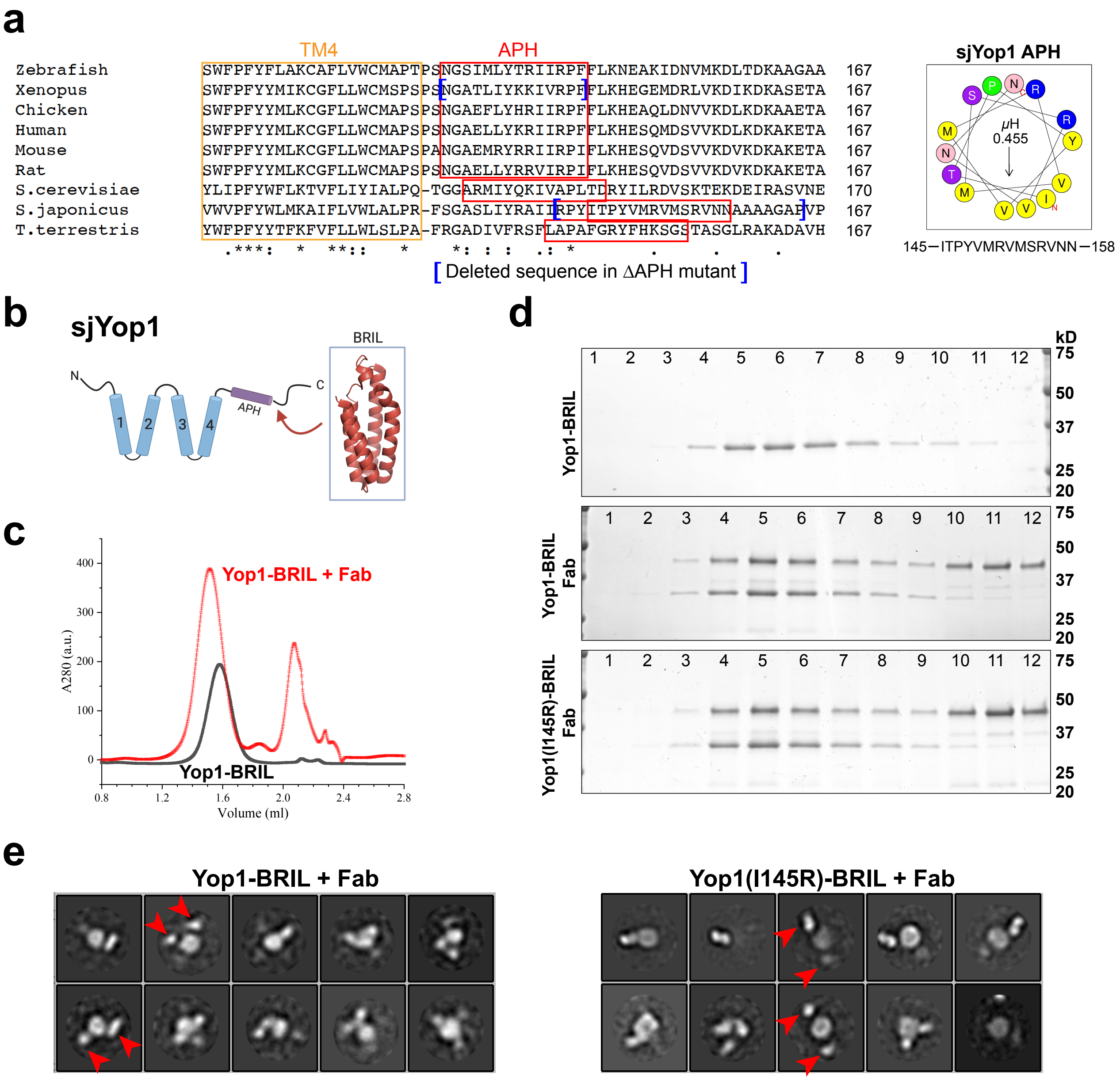

Supplementary Figure 1. REEPs form stable dimers. a, Sequence alignment of REEPs from the indicated species was performed with Clustal Omega. The last TM (TM4) and the APHs are indicated by yellow and red boxes, respectively. Brackets show the regions deleted in REEP5ΔAPH and Yop1ΔAPH. The right panel shows a helical wheel representation of the APH of Yop1, as determined with HELIQUEST. Hydrophobic residues are shown in yellow, positively charged residues in blue, hydrophilic, uncharged residues in pink and purple, and a Pro residue in green. The calculated hydrophobic moment (μH) is indicated. **b**, Scheme of the fusion of Yop1 and BRIL. **c**, The Yop1-BRIL fusion protein was purified from DDM-solubilized membranes and incubated with purified Fabs directed against BRIL. The sample was subjected to SEC (red trace of absorbance at 280 nm). As a control, Yop1-BRIL was analyzed without Fabs (black trace). **d**, Fractions of the SEC runs were analyzed by SDS-PAGE and Coomassie-blue staining. A Yop1-BRIL fusion with a mutation in the APH (I145K) was also incubated with Fabs and analyzed. **e**, Negative-stain EM analysis of the indicated samples. Shown are 2D class averages with bound Fabs indicated by arrowheads. These experiments were repeated once.

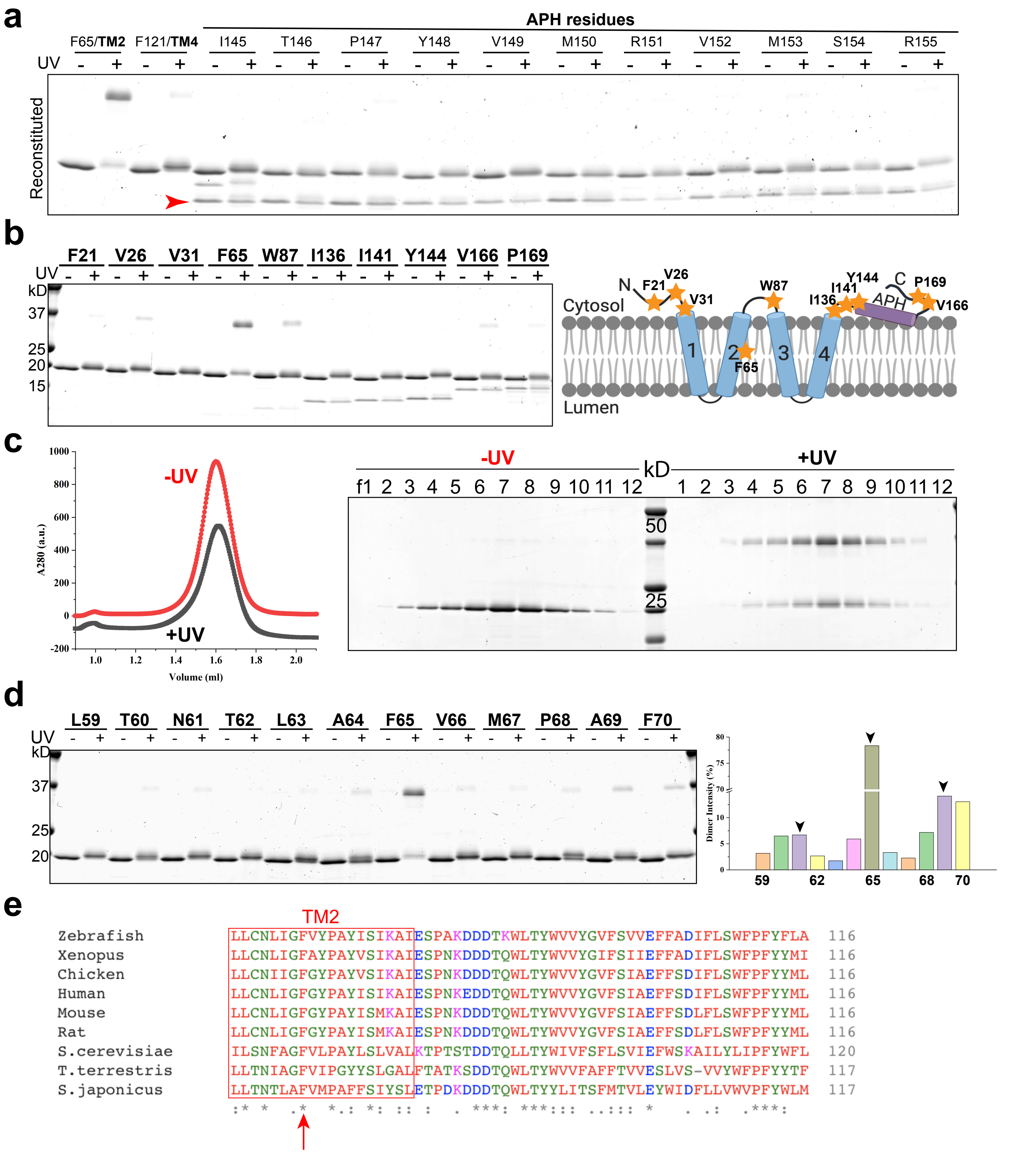

Supplementary Figure 2. REEP monomers form dimers through interaction of TM2. **a**, Photoreactive Bpa probes were incorporated into Yop1 at the indicated positions by amber codon suppression. The proteins were purified and reconstituted into liposomes containing *S. cerevisiae* lipids. The samples were irradiated with UV light, as indicated, and analyzed by SDS-PAGE and Coomassie-blue staining. The arrowhead indicates the position of C-terminally truncated proteins, generated by premature termination of translation at the introduced amber codons. Note that these fragments co-purify with the full-length proteins, confirming that dimerization does not require the APH domain. The experiment was repeated twice. **b**, As in **a**, but with Bpa in the N-terminal segment (F21, V26, V31), the cytosolic loop between TM2 and TM3 (W87), the unstructured segment preceding the APH (I136, I141, Y144), or the C-terminal segment (V166, P169) (see scheme on the right). The experiment was repeated once. **c**, Purified SBP-tagged Yop1F65Bpa was reconstituted into liposomes and irradiated with UV light. A control sample was not irradiated. Proteins of both samples were solubilized with 1% DDM, bound to streptavidin beads, and eluted by proteolytic removal of the SBP tag. The proteins were then subjected to SEC. The elution profiles (absorbance at 280 nm) are shown on the left, and the analysis of fractions by SDS-PAGE and Coomassie-blue staining on the right. The experiment was repeated once. **d**, As in **a**, but with probes at different positions of TM2. The percentage of dimer formation was quantified for each position (right panel). Crosslinking yields show peaks that are ~4 residues apart, as expected for an interaction of the TM2 helices of the two monomers. The experiment was repeated once. **e**, Sequence alignment of REEPs, showing the conserved Phe residue in TM2 (arrow). TM2 is indicated by a red box.

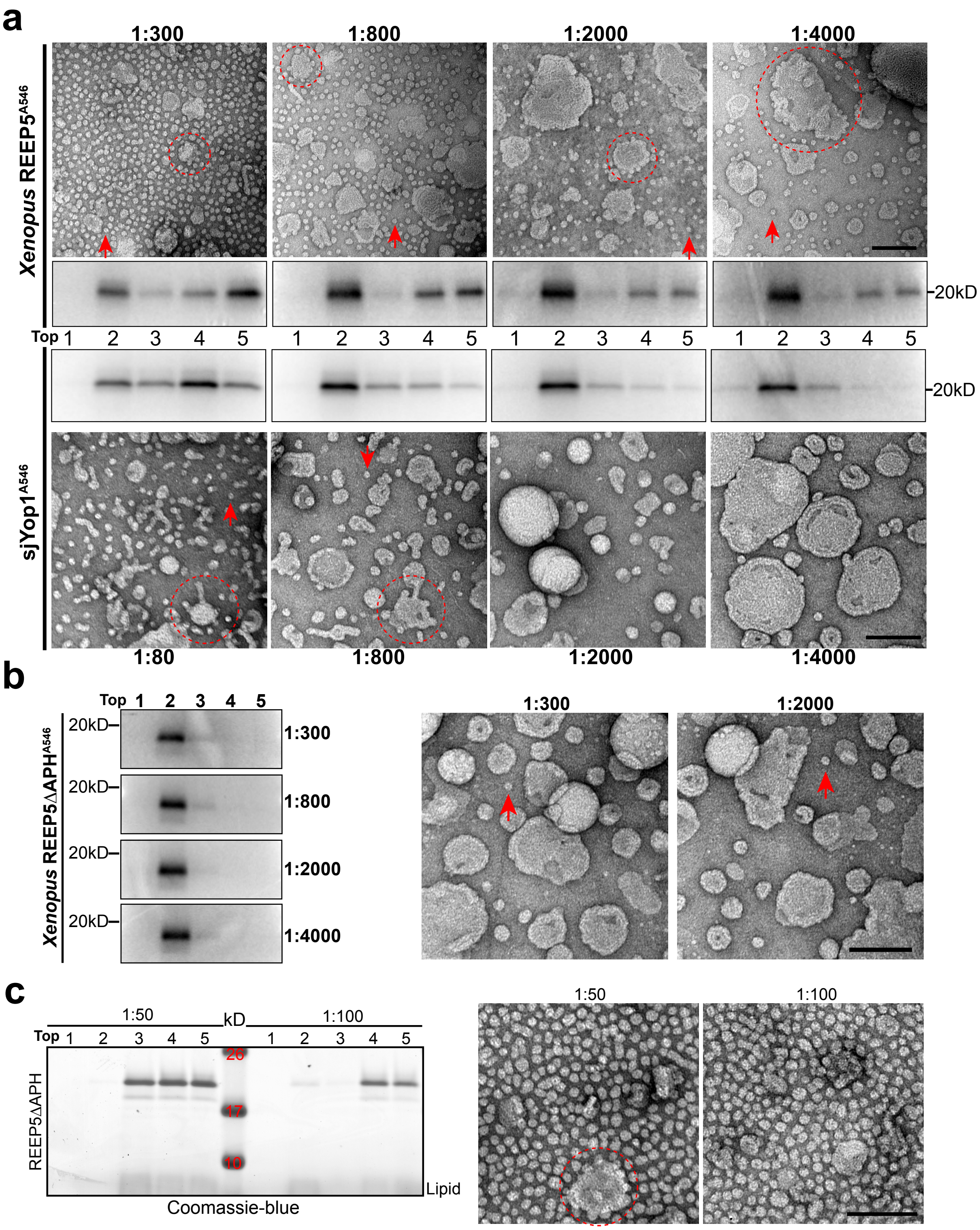

Supplementary Figure 3. REEPs form LPPs in a reconstituted system. a, Wild-type *Xenopus* REEP5 or Yop1, both labeled with Alexa 546, were reconstituted with *S. cerevisiae* lipids at the indicated protein to lipid ratios. The samples were analyzed by negative-stain EM. Arrows point to LPPs with a size of ~8 nm. Dashed circles indicate liposomes from which LPPs (REEP5) or short tubules (Yop1) bud off. Bars, 100 nm. All samples were also subjected to flotation in a Nycodenz gradient. Fractions were analyzed with a fluorescence scanner. **b**, As in **a**, but for *Xenopus* REEP5 lacking the APH (REEP5ΔAPH). **c**, As in **b**, but with higher concentrations of REEP5ΔAPH and analysis of the Nycodenz gradient fractions by Coomassie-blue staining. All these experiments were repeated once.

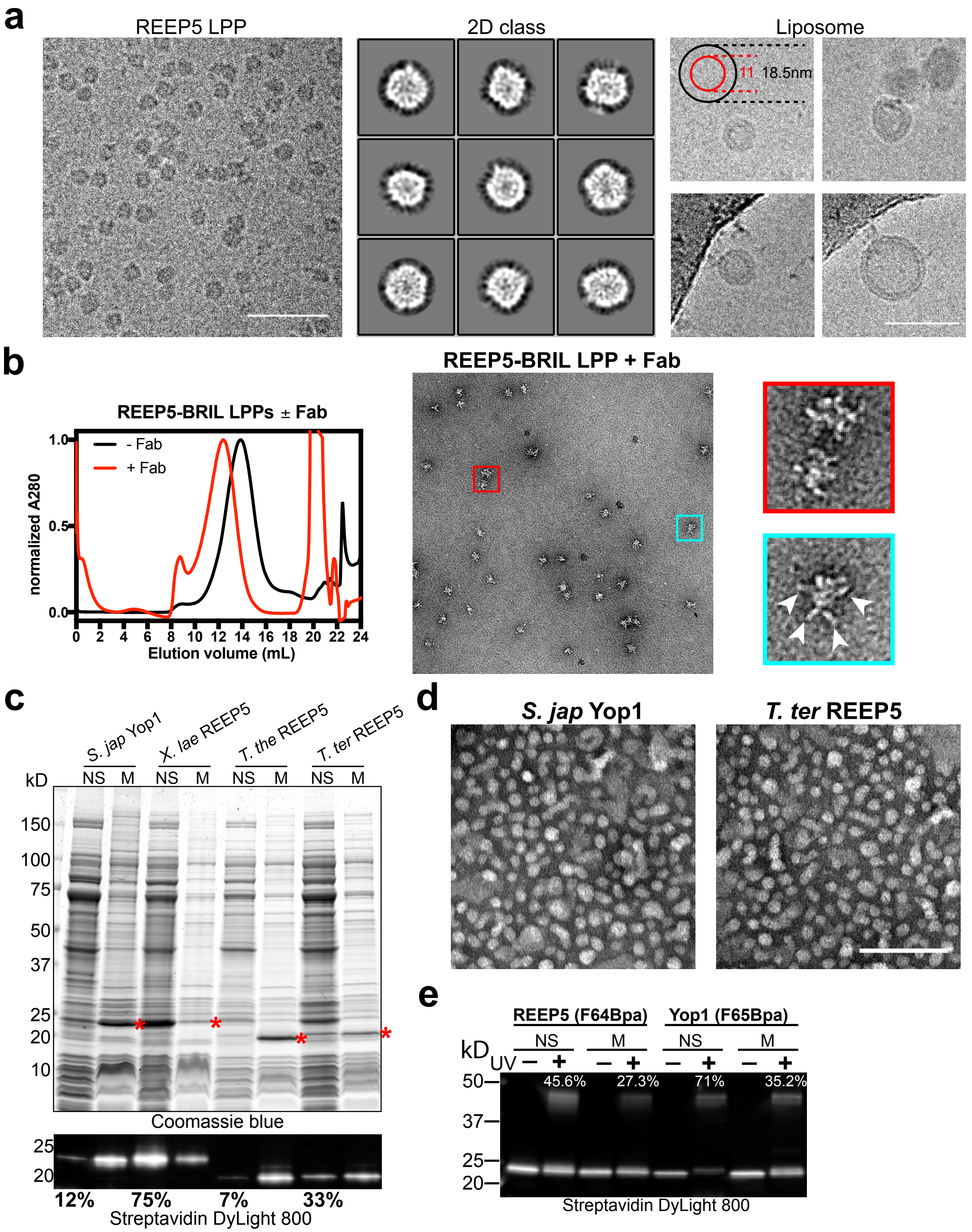

Supplementary Figure 4. REEPs form LPPs when overexpressed in *E.coli*. a, *Xenopus* REEP5 was expressed in *E. coli* and the generated LPPs were purified and analyzed by cryo-EM. The middle panel shows 2D average classes. Note that no phospholipid bilayer is visible. The right panel shows protein-free liposomes, generated by sonication. The highlighted liposome has diameters of the inner and outer layers (11 and 18.5 nm) corresponding to a lipid bilayer thickness of 3.75 nm. Bars, 50 nm. **b**, REEP5-BRIL was expressed in *E. coli* and the LPPs were purified. They were either incubated with Fabs directed against BRIL or left untreated. Both samples were subjected to SEC. Shown are the traces at 280 nm, normalized to the maximum absorbance (upper left panel). Fractions were analyzed by negative-stain EM (right panel). Red and blue boxes are shown in magnified views. They show examples of multiple Fabs (arrowheads) bound to a LPP. The experiment was repeated once. **c**, SBP-tagged Yop1 from *Schizosaccharomyces japonicus* (*S. jap* Yop1), or SBP-tagged REEP5 from *Xenopus laevis* (*X. lae* REEP5), *Thermothelomyces thermophila* (*T. the* REEP5) or *Thielavia terrestris* (*T. ter* REEP5) were expressed in *E. coli*. Lysates were separated by centrifugation into a non-sedimentable (NS) fraction (LPPs) and a membrane (M) fraction. The samples were analyzed by SDS-PAGE and Coomassie-blue staining (upper panel) and by blotting for the SBP-tag with Dylight 800-labeled streptavidin (lower panel). Asterisks indicate the positions of the REEP5 proteins. The percentage of protein in the NS fraction was quantified from the streptavidin blot and is shown at the bottom. The experiment was repeated once. **d**, Negative-stain images of *S. jap* Yop1 and *T. ter* REEP5 LPPs. The samples were analyzed before SEC. Bar, 100 nm. These experiments were repeated once. **e**, SBP-tagged REEP5F64Bpa or Yop1F65Bpa were expressed in *E. coli*. Lysates were separated by centrifugation into NS and M fractions. Both fractions were subjected to UV irradiation, as indicated, and analyzed by SDS-PAGE and blotting with Dylight 800-labeled streptavidin. The relative intensity of dimer crosslinks was quantified and is shown above the bands. The experiment was repeated twice.

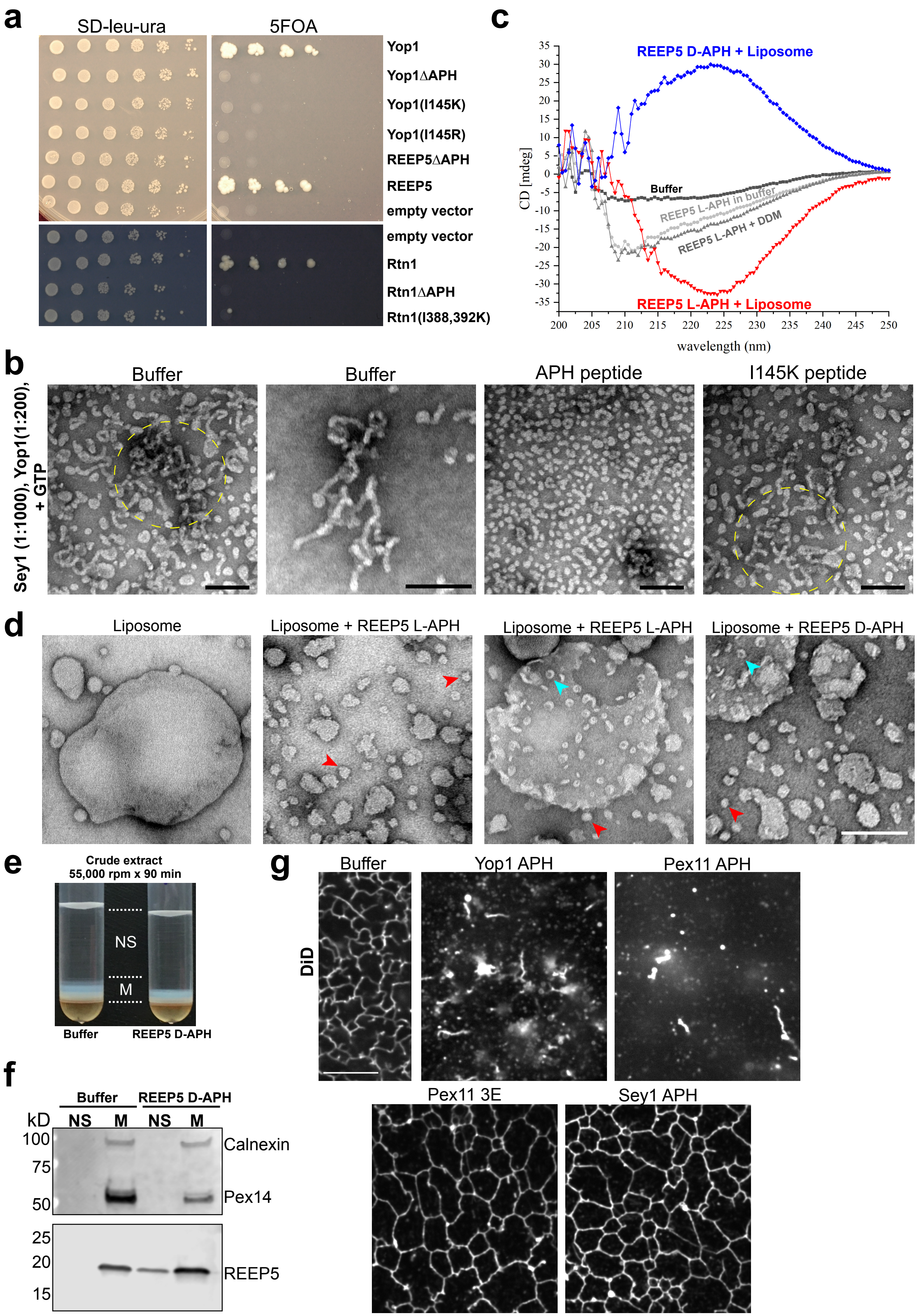
a
SD-leu-ura
5FOA
Yop1
Yop1ΔAPH
Yop1(I145K)
Yop1(I145R)
REEP5ΔAPH
REEP5
empty vector
empty vector
Rtn1
Rtn1ΔAPH
Rtn1(I388,392K)
c
REEP5 D-APH + Liposome
Buffer
REEP5 L-APH in buffer
REEP5 L-APH + DDM
REEP5 L-APH + Liposome
CD [mdeg]
wavelength (nm)
b
Sey1 (1:1000), Yop1(1:200), + GTP
Buffer
Buffer
APH peptide
I145K peptide
d
Liposome
Liposome + REEP5 L-APH
Liposome + REEP5 L-APH
Liposome + REEP5 D-APH
e
Crude extract
55,000 rpm x 90 min
NS
M
Buffer
REEP5 D-APH
f
Buffer
REEP5 D-APH
kD
NS
M
NS
M
100
75
50
Calnexin
Pex14
25
20
15
REEP5
g
DiD
Buffer
Yop1 APH
Pex11 APH
Pex11 3E
Sey1 APH

Supplementary Figure 5. The APH of REEPs generates high membrane curvature. a, *S. cerevisiae* cells containing triple knock-outs of *rtn1*, *yop1* and *nup85* were transformed with a centromeric plasmid expressing wild-type *S. japonicus* Yop1, *S. japonicus* Rtn1, or *Xenopus* REEP5 under the native promoter of *S. cerevisiae* Yop1. Similar experiments were done with plasmids coding for proteins with mutations in the APH. Cells were serially diluted and plated without leucine and uracil on plates lacking or containing 5FOA to cure the Nup85-encoding plasmid required for cell viability. The plates were incubated at 30°C in the dark for 4 days before taking images. **b**, Yop1 and the GTPase Sey1 were co-reconstituted into liposomes containing *S. cerevisiae* lipids. The samples were incubated with $MgCl_2$ and GTP for 10 min before addition of the indicated peptides (1:20 molar ratio of peptide to lipid). Controls were incubated with buffer lacking peptides. All samples were analyzed by negative-stain EM. Although negative-staining affects the integrity of the tubular network, some interconnected tubules are visible in the control and after addition of mutant peptide (dashed circles). Bars, 100 nm. **c**, The indicated peptides were analyzed by CD spectroscopy in buffer, DDM, or after incubation with liposomes. The sequences of the peptides are listed in Supplementary Table 3. **d**, Non-extruded, protein-free liposomes generated with *S. cerevisiae* lipids were incubated with the synthetic peptides REEP5 L-APH or REEP5 D-APH at 1:20 molar ratio of peptide to lipid and analyzed by negative-stain EM. Small liposomes generated by the peptides are indicated by red arrowheads. Small vesicles or LPPs on the surface of larger liposomes are indicated by blue arrowheads; they might be in the process of budding off. Bar, 100 nm. **e**, *Xenopus* egg interphase extract was diluted 1:1 with buffer and incubated at room temperature with or without REEP5 D-APH peptide at a final concentration of 100 μM. The samples were subjected to centrifugation at 248,000 g for 90 min to separate membranes (M) from a non-sedimentable (NS) fraction. **f**, The membranes were resuspended in buffer to the volume of the NS fraction. Aliquots were subjected to SDS-PAGE and blotting with antibodies to *Xenopus* REEP5. Other aliquots were used for immunoblotting for calnexin and Pex14 as membrane markers. **g**, An interphase ER network was generated with *Xenopus* egg extract in the presence of buffer or 100 μM of the APH peptides of *Penicillium chrysogenum* Pex11 (Pex11 APH), a mutant APH containing three Glu residues (Pex11 3E), or the APH of *S. japonicus* Sey1(Sey1 APH). All samples were stained with the fluorescent dye DiD and visualized in a fluorescence microscope. Bar, 10 μm. All the experiments were repeated at least once.

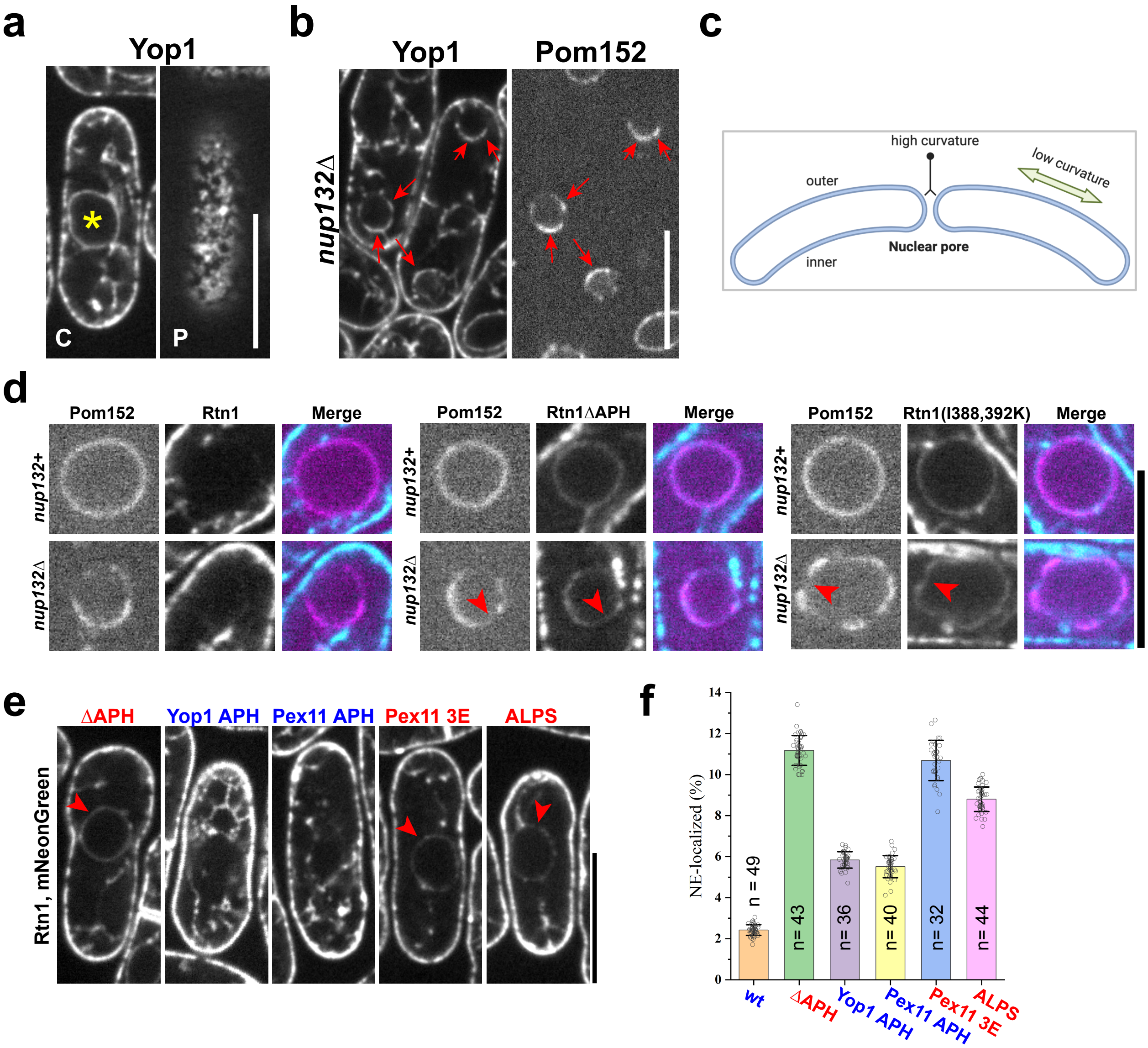

Supplementary Figure 6. Localization of wild-type and mutant Yop1 and Rtn1 in *S. japonicus* cells. **a**, Yop1-mNeonGreen was expressed in *S. japonicus* cells from the native genomic locus. The cells were analyzed in a confocal fluorescence microscope. Shown are images focused on the center (C) or periphery (P) of the cell. The nucleus is marked with an asterisk. **b**, As in **a**, but with cells lacking Nup132, in which nuclear pores are clustered, as shown by the localization of the pore marker Pom152. Note that Yop1-mNeonGreen also clusters (arrows), indicating that it is localized to the pores. Bars, 10 µm. These experiments were repeated twice. **c**, The scheme illustrates the high membrane curvature at nuclear pores, and the low curvature elsewhere in the nuclear envelope. **d**, mNeonGreen-tagged versions of wild-type or mutant Rtn1 were expressed in *nup132Δ* cells. Note that Rtn1 with APH mutations does not co-cluster with Pom152. Arrowheads point to regions containing Rtn1 mutants but not Pom152. Bar, 10 µm. The experiment was repeated twice. **e**, Localization of mNeonGreen-tagged mutants of Rtn1 in *S. japonicus* cells. The APH was either deleted or replaced by the APHs of Yop1, Pex11, a mutant of Pex11 (Pex11 3E), or the ALPS sequence of rat ArfGAP1. Shown are images focused on the center of the cells. Arrowheads point to the nuclear envelope (NE). Bars, 10 µm. **f**, Quantification of experiments shown in **e**. The mean percentages of fluorescent protein in the NE is shown together with SD and the number of cells analyzed from two independent experiments. Rtn1 mutants labeled in red have a defect in the exclusive localization to the peripheral ER.

Supplementary Table 1. Plasmids used in this study

Plasmid	Description	Gene/Mutation
NWP63	pET21b-Yop1-3C-His10	*S. japonicus* Yop1/wt
NWP150	pET21b-Yop1(Δ142-165)-3C-His10	*S. japonicus* Yop1/lacking APH
NWP207	pET21b-Yop1-3C-SBP	*S. japonicus* Yop1/wt
NWP322	pET28-Yop1-TEV-SBP	*S. japonicus* Yop1/wt
NWP323	pET28-Yop1(Δ142-165)-TEV-SBP	*S. japonicus* Yop1/lacking APH
NWP118	pET28-REEP5(*X. lae*)-TEV-SBP	*X. laevis* REEP5/wt
NWP225	pET21b-Yop1(F47Bpa)-3C-SBP	*S. japonicus* Yop1/codon encoding F47 mutated to amber codon TAG (F47Bpa)
NWP226	pET21b-Yop1(F65Bpa)-3C-SBP	*S. japonicus* Yop1/F65Bpa
NWP227	pET21b-Yop1(F96Bpa)-3C-SBP	*S. japonicus* Yop1/F96Bpa
NWP228	pET21b-Yop1(F121Bpa)-3C-SBP	*S. japonicus* Yop1/F121Bpa
NWP294	pET21b-Yop1(F65Bpa, I145R)-3C-SBP	*S. japonicus* Yop1/F65Bpa, I145R
NWP295	pET21b-Yop1(F65Bpa, I145K)-3C-SBP	*S. japonicus* Yop1/F65Bpa, I145K
NWP296	pET21b-Yop1(F65Bpa, I145E)-3C-SBP	*S. japonicus* Yop1/ F65Bpa, I145E
NWP120	pET21b-REEP5(*T. the*)-3C-SBP	*T. thermophila* REEP5/wt
NWP121	pET21b-REEP5(*T. ter*)-3C-SBP	*T. terrestris* REEP5/wt
NWP309	pET28-REEP5((*X. lae,* F64Bpa)-TEV-SBP	*X. laevis* REEP5/ F64Bpa
NWP310	pET21b-REEP5(*T. the*, F66Bpa)-3C-SBP	*T. thermophila* REEP5/F66Bpa
NWP311	pET21b-REEP5(*T. ter*, F66Bpa)-3C-SBP	*T. terrestris* REEP5/F66Bpa
NWP165	pET28-Sey1(*S. cer*)-TEV-SBP	*S. cerevisiae* Sey1/wt
NWP137	pET28-REEP5(*X. lae*, Δ131-144)-TEV-SBP	*X. laevis* REEP5/lacking APH
NWP115	pET21b-Yop1(I145S)-3C-His10	*S. japonicus* Yop1/I145S
NWP87	pET21b-Yop1(I145K)-3C-His10	*S. japonicus* Yop1/ I145K
NWP176	pET21b-Yop1(1-144)-Rtn1(379-392)-Yop1(159-191)-3C-His10	*S. japonicus* Yop1/the APH was replaced with sjRtn1's APH
NWP281	pET21b-Yop1(1-173)-BRIL-(174-191)-3C-SBP	*S. japonicus* Yop1/BRIL was inserted between aa173 and 174.
NWP299	pET21b-Yop1(1-173, I145R)-BRIL-(174-191)-3C-SBP	*S. japonicus* Yop1/ BRIL was inserted between aa173 and 174, I145 in Yop1 APH was mutated to R
NWP214	pET21b-Yop1(I145Bpa)-3C-His10	*S. japonicus* Yop1/I145Bpa
NWP215	pET21b-Yop1(T146Bpa)-3C-His10	*S. japonicus* Yop1/T146Bpa
NWP216	pET21b-Yop1(P147Bpa)-3C-His10	*S. japonicus* Yop1/P147Bpa
NWP217	pET21b-Yop1(Y148Bpa)-3C-His10	*S. japonicus* Yop1/Y148Bpa
NWP218	pET21b-Yop1(V149Bpa)-3C-His10	*S. japonicus* Yop1/V149Bpa
NWP219	pET21b-Yop1(M150Bpa)-3C-His10	*S. japonicus* Yop1/M150Bpa
NWP233	pET21b-Yop1(R151Bpa)-3C-SBP	*S. japonicus* Yop1/R151Bpa
NWP234	pET21b-Yop1(V152Bpa)-3C-SBP	*S. japonicus* Yop1/V152Bpa
NWP236	pET21b-Yop1(M153Bpa)-3C-SBP	*S. japonicus* Yop1/M153Bpa

NWP237	pET21b-Yop1(S154Bpa)-3C-SBP	*S. japonicus* Yop1/S154Bpa
NWP238	pET21b-Yop1(R155Bpa)-3C-SBP	*S. japonicus* Yop1/R155Bpa
NWP332	pET21b-Yop1(F21Bpa)-3C-SBP	*S. japonicus* Yop1/F21Bpa
NWP333	pET21b-Yop1(V26Bpa)-3C-SBP	*S. japonicus* Yop1/V26Bpa
NWP334	pET21b-Yop1(V31Bpa)-3C-SBP	*S. japonicus* Yop1/V31Bpa
NWP338	pET21b-Yop1(W87Bpa)-3C-SBP	*S. japonicus* Yop1/W87Bpa
NWP272	pET21b-Yop1(I136Bpa)-3C-SBP	*S. japonicus* Yop1/I136Bpa
NWP273	pET21b-Yop1(I141Bpa)-3C-SBP	*S. japonicus* Yop1/I141Bpa
NWP274	pET21b-Yop1(Y144Bpa)-3C-SBP	*S. japonicus* Yop1/Y144Bpa
NWP275	pET21b-Yop1(V166Bpa)-3C-SBP	*S. japonicus* Yop1/V166Bpa
NWP336	pET21b-Yop1(P169Bpa)-3C-SBP	*S. japonicus* Yop1/P169Bpa
NWP321	pET21b-Yop1(L59Bpa)-3C-SBP	*S. japonicus* Yop1/L59Bpa
NWP320	pET21b-Yop1(T60Bpa)-3C-SBP	*S. japonicus* Yop1/T60Bpa
NWP304	pET21b-Yop1(N61Bpa)-3C-SBP	*S. japonicus* Yop1/N61Bpa
NWP303	pET21b-Yop1(T62Bpa)-3C-SBP	*S. japonicus* Yop1/T62Bpa
NWP287	pET21b-Yop1(L63Bpa)-3C-SBP	*S. japonicus* Yop1/L63Bpa
NWP288	pET21b-Yop1(A64Bpa)-3C-SBP	*S. japonicus* Yop1/A64Bpa
NWP289	pET21b-Yop1(V66Bpa)-3C-SBP	*S. japonicus* Yop1/V66Bpa
NWP290	pET21b-Yop1(M67Bpa)-3C-SBP	*S. japonicus* Yop1/M67Bpa
NWP291	pET21b-Yop1(P68Bpa)-3C-SBP	*S. japonicus* Yop1/P68Bpa
NWP292	pET21b-Yop1(A69Bpa)-3C-SBP	*S. japonicus* Yop1/A69Bpa
NWP293	pET21b-Yop1(F70Bpa)-3C-SBP	*S. japonicus* Yop1/F70Bpa

Supplementary Table 2. Primers used in this study

Primer name	Sequence
Yop1(I145K) F	GCAATTATTCGTCCGTATAAAACCCCGTATGTTATG
Yop1(I145K) R	TTTATACGGACGAATAATTGCACGATAAATCAGGCT
Yop1(I145R) F	GCAATTATTCGTCCGTATCGUACCCCGTATGTTATG
Yop1(I145R) R	ACGATACGGACGAATAATTGCACGATAAATCAGGCT
Yop1(I145E) F	GCAATTATTCGTCCGTATGAAACCCCGTATGTTATG
Yop1(I145E) R	TTCATACGGACGAATAATTGCACGATAAATCAGGCT
Yop1(Δ142-165) F	TATCGTGCAATTATTGTTCCGCCTCCGCGT
Yop1(Δ142-165) R	AATAATTGCACGATAAATCAGGCTTGCACC
Yop1(F47Bpa) F	TAGGCACTGCTGTTTCTGAATTTTGGT
Yop1(F47Bpa) R	AAACAGCAGTGCCTAATAAACGGTGGCTAA
Yop1(F65Bpa) F	TAGGTTATGCCTGCCTTTTTCAGC
Yop1(F65Bpa) R	GGCAGGCATAACCTATGCCAGTGTATTGGT
Yop1(F96Bpa) F	TAGATGACCGTGCTGGAATATTGG
Yop1(F96Bpa) R	CAGCACGGTCATCTAGCTGGTAATCAGATA
Yop1(F121Bpa) F	TAGCTGGTGTGGCTGGCACTGCCT
Yop1(F121Bpa) R	CAGCCACACCAGCTAAATTGCTTTCATCAG
REEP5(*X.lae*, Δ131-144) R	CTCATGCTTTAGGAAGGAGGGGCTGGGAGA
REEP5(*X.lae*, Δ131-144) F	TTCCTAAAGCATGAGGGGGAGATG
Yop1APHswapRtn1 R	CTTAATGTTGGACAAGGTGCCGTCAATGACGCGCTTG TTCGCAATATTGCACGATAAAT
Yop1APHswapRtn1 F	TCCAACATTAAGTTTCAGATCGAGGAATATACATGCC AATTT GTTCCGCCTCCGCGTAGC
Yop1(I145Bpa) F	TAGACCCCGTATGTTATGCGTGTTATGAGC
Yop1(I145Bpa) R	AACATACGGGGTCTAATACGGACGAATAATTGC
Yop1(T146Bpa) F	TAGCCGTATGTTATGCGTGTTATGAGCCGT
Yop1(T146Bpa) R	CATAACATACGGCTAGATATACGGACGAATAAT
Yop1(P147Bpa) F	TAGTATGTTATGCGTGTTATGAGCCGTGTT
Yop1(P147Bpa) R	ACGCATAACATACTAGGTGATATACGGACGAAT
Yop1(Y148Bpa) F	TAGGTTATGCGTGTTATGAGCCGTGTTAAT
Yop1(Y148Bpa) R	AACACGCATAACCTACGGGGTGATATACGGACG
Yop1(V149Bpa) F	TAGATGCGTGTTATGAGCCGTGTTAATAAT
Yop1(V149Bpa) R	CATAACACGCATCTAATACGGGGTGATATACGG
Yop1(M150Bpa) F	TAGCGTGTTATGAGCCGTGTTAATAATGCA
Yop1(M150Bpa) R	GCTCATAACACGCTAAACATACGGGGTGATATA
Yop1(R151Bpa) F	TAGGTTATGAGCCGTGTTAATAATGCAGCC
Yop1(R151Bpa) R	ACGGCTCATAACCTACATAACATACGGGGTGAT
Yop1(V152Bpa) F	TAGATGAGCCGTGTTAATAATGCAGCCGCA
Yop1(V152Bpa) R	AACACGGCTCATCTAACGCATAACATACGGGGT
Yop1(M153Bpa) F	TAGAGCCGTGTTAATAATGCAGCCGCAGCG
Yop1(M153Bpa) R	ATTAACACGGCTCTAAACACGCATAACATACGG
Yop1(S154Bpa) F	TAGCGTGTTAATAATGCAGCCGCAGCGGGT
Yop1(S154Bpa) R	ATTATTAACACGCTACATAACACGCATAACATA

Yop1(R155Bpa) F	TAGGTTAATAATGCAGCCGCAGCGGGTGCA
Yop1(R155Bpa) R	TGCATTATTAACCTAGCTCATAACACGCATAAC
Yop1(F21Bpa) F	AAACGTCTGAGCGCATAGCCGGTTCTGAATGTT
Yop1(F21Bpa) R	CTATGCGCTCAGACGTTTATCCAGAAC
Yop1(V26Bpa) F	TTTCCGGTTCTGAATTAGATGGAACAGAAAGTG
Yop1(V26Bpa) R	CTAATTCAGAACCGGAAATGCGCTCAG
Yop1(V31Bpa) F	GTTATGGAACAGAAATAGGGTGTGAACAAAGTG
Yop1(V31Bpa) R	CTATTTCTGTTCCATAACATTCAG
Yop1(W87Bpa) F	GATGATGATACCCAGTAGCTGACCTATTAT
Yop1(W87Bpa) R	CTACTGGGTATCATCATCTTTATC
Yop1(I136Bpa) F	TAGTATCGTGCAATTATTCGT
Yop1(I136Bpa) R	AATTGCACGATACTACAGGCTTGCACCGCT
Yop1(I141Bpa) F	TAGCGTCCGTATATCACCCCG
Yop1(I141Bpa) R	GATATACGGACGCTAAATTGCACGATAAAT
Yop1(Y144Bpa) F	TAGATCACCCCGTATGTTATGCGT
Yop1(Y144Bpa) R	ATACGGGGTGATCTACGGACGAATAATTGC
Yop1(V166Bpa) F	TAGCCGCCTCCGCGTAGCGAA
Yop1(V166Bpa) R	ACGCGGAGGCGGCTACGGTGCACCCGCTGC
Yop1(P169Bpa) F	GCACCGGTTCCGCCTTAGCGTAGCGAAAAACCG
Yop1(P169Bpa) R	CTAAGGCGGAACCGGTGCACCCGCTGC
Yop1(L59Bpa) F	TAGACCAATACACTGGCATTTGTT
Yop1(L59Bpa) R	CAGTGTATTGGTCTACAGATAACCACC
Yop1(T60Bpa) F	TAGAATACACTGGCATTTGTTATG
Yop1(T60Bpa) R	TGCCAGTGTATTCTACAGCAGATAACC
Yop1(N61Bpa) F	TAGACACTGGCATTTGTTATGCCT
Yop1(N61Bpa) R	AAATGCCAGTGTCTAGGTCAGCAGATA
Yop1(T62Bpa) F	TAGCTGGCATTTGTTATGCCTGCC
Yop1(T62Bpa) R	AACAAATGCCAGCTAATTGGTCAGCAG
Yop1(L63Bpa) F	TAGGCATTTGTTATGCCTGCCTTT
Yop1(L63Bpa) R	CATAACAAATGCCTATGTATTGGTCAGCAG
Yop1(A64Bpa) F	TAGTTTGTTATGCCTGCCTTTTTC
Yop1(A64Bpa) R	AGGCATAACAAACTACAGTGTATTGGTCAG
Yop1(V66Bpa) F	TAGATGCCTGCCTTTTTCAGCATT
Yop1(V66Bpa) R	AAAGGCAGGCATCTAAAATGCCAGTGTATT
Yop1(M67Bpa) F	TAGCCTGCCTTTTTCAGCATTTAT
Yop1(M67Bpa) R	GAAAAAGGCAGGCTAAACAAATGCCAGTGT
Yop1(P68Bpa) F	TAGGCCTTTTTCAGCATTTATAGC
Yop1(P68Bpa) R	GCTGAAAAAGGCCTACATAACAAATGCCAG
Yop1(A69Bpa) F	TAGTTTTTCAGCATTTATAGCCTG
Yop1(A69Bpa) R	AATGCTGAAAAACTAAGGCATAACAAATGC
Yop1(F70Bpa) F	TAGTTCAGCATTTATAGCCTGGAA
Yop1(F70Bpa) R	ATAAATGCTGAACTAGGCAGGCATAACAAA

Supplementary Table 3. Synthetic peptide sequence

Peptide name	Sequence
Yop1 APH	SLIYRAIIRPYITPYVMRVMSRVNNAAAAGAPVPPPR
Yop1 I145K	SLIYRAIIRPYKTPYVMRVMSRVNNAAAAGAPVPPPR
REEP5 APH	SNGATLIYKKIVRPFFLKHEG
Pex11 APH	YNAVKKQFGTTRKIMRIGKFLEHLKAAAVAF
Pex11 3E	YNAVKKQFGTTRKEMREGKELEHLKAAAVAF
Sey1 APH	AGPVKAFAERSVRNAVNSMGEKLAEKLDDYRSTSPA

Supplementary Table 4. ***S. japonicus*** **strains used in this study**

Name	Genotype
NWY195	*rtn1-mNeonGreen-kanMX6 pom152-mCherry-hphMX6 ura4-D3 ade6-domK*
NWY309	*rtn1(Δ379-396)-mNeonGreen-kanMX6 pom152-mCherry-hphMX6 ura4-D3 ade6*
NWY310	*rtn1(I388,392K)-mNeonGreen-kanMX6 pom152-mCherry-hphMX6 ura4-D3 ade6*
NWY49	*h^- rtn1-mNeonGreen-kanMX6 ura4-D3 ade6-domK*
NWY247	*h^- rtn1(Δ379-396)-mNeonGreen-kanMX6 ura4-D3 ade6-domK*
NWY47	*h^- yop1-mNeonGreen-kanMX6 ura4-D3 ade6-domK*
NWY279	*h^- yop1(I145K)-mNeonGreen-kanMX6 ura4-D3 ade6-domK*
NWY194	*nup132Δ::natMX6 yop1-mNeonGreen-kanMX6 pom152-mCherry-kanMX6 ura4-D3 ade6*
NWY196	*nup132Δ::natMX6 rtn1-mNeonGreen-kanMX6 pom152-mCherry-hphMX6 ura4-D3 ade6*
NWY303	*nup132Δ::natMX6 rtn1(Δ379-396)-mNeonGreen-kanMX6 pom152-mCherry-hphMX6 ura4-D3 ade6*
NWY304	*nup132Δ::natMX6 rtn1(I388,392K)-mNeonGreen-kanMX6 pom152-mCherry-hphMX6 ura4-D3 ade6*
NWY363	*rtn1(1-378)-yop1(145-162)-rtn1(397-425)-mNeonGreen-kanMX6 ura4-D3 ade6-domK*
NWY368	*h^- rtn1(1-378)-pex11APH(66-83)-rtn1(397-420)-mNeonGreen-kanMX6 ura4-D3 ade6-domK*
NWY370	*h^- rtn1(1-378)-pex11APH(66-83, I69E, I72E, F75E)-rtn1(397-420)-mNeonGreen-kanMX6 ura4-D3 ade6-domK*
NWY369	*h^- rtn1(1-378)-ArfGAP1APH(199-223)- rtn1(397-420)-mNeonGreen-kanMX6 ura4-D3 ade6-domK*
